# DNA methylation as a triage tool for cervical cancer screening – A meeting report

**DOI:** 10.1016/j.pmedr.2024.102678

**Published:** 2024-03-06

**Authors:** F. Ricardo Burdier, Dur-e-Nayab Waheed, Belinda Nedjai, Renske D.M. Steenbergen, Mario Poljak, Marc Baay, Alex Vorsters, Severien Van Keer

**Affiliations:** a Centre for the Evaluation of Vaccination, Vaccine and Infectious Disease Institute, University of Antwerp, Belgium; b Centre for Prevention, Diagnosis and Detection Cancer Prevention Unit, Wolfson Institute of Population Health, Queen Mary University of London, London, UK; c Amsterdam UMC Vrije Universiteit Amsterdam, Department of Pathology, Amsterdam, The Netherlands; d Cancer Center Amsterdam, Imaging and Biomarkers, Amsterdam, The Netherlands; e Institute of Immunology and Microbiology, Faculty of Medicine, University of Ljubljana, Ljubljana, Slovenia; f P95 Epidemiology & Pharmacovigilance, Leuven, Belgium

**Keywords:** Cervical cancer screening, Human papillomavirus infection, Prognostic biomarker, Triage, Methylation, Self-sampling, Molecular biomarker, HPV-related disease

## Abstract

**Introduction:**

DNA methylation is proposed as a novel biomarker able to monitor molecular events in human papillomavirus (HPV) infection pathophysiology, enabling the distinction between HPV-induced lesions with regression potential from those that may progress to HPV-related cancer.

**Methods:**

This meeting report summarises the presentations and expert discussions during the HPV Prevention and Control Board-focused topic technical meeting on DNA methylation validation in clinician-collected and self-collected samples, novel DNA methylation markers discovery, implementation in cervical cancer screening programs, and their potential in women living with human immunodeficiency virus (HIV).

**Results:**

Data presented in the meeting showed that HPV-positive, baseline methylation-negative women have a lower cumulative cervical cancer incidence than baseline cytology-negative women, making DNA methylation an attractive triage strategy. However, additional standardised data in different settings (low- versus high-income settings), samples (clinician-collected and self-collected), study designs (prospective, modelling, impact) and populations (immunocompetent women, women living with HIV) are needed.

**Conclusion:**

Establishing international validation guidelines were identified as the way forward towards accurate validation and subsequent implementation in current screening programs.

## 1 Introduction

The HPV Prevention and Control Board is an independent, international, and multidisciplinary group of experts, created in 2015 to provide evidence-based guidance and reflection on strategic, technical, and policy issues regarding implementation and sustainability of human papillomavirus (HPV) prevention and control programmes. The board aims to disseminate and amplify relevant information on HPV prevention and control to a broad array of stakeholders by organising two meetings every year; a “technical meeting”, covering practical and technological topics such as vaccine characteristics, vaccine safety, screening strategies and technologies landscape, treatment approaches, the role of healthcare providers in vaccination programmes, and dealing with anti-vaccine messages (Vorsters et al., 2017, Vorsters et al., 2019, D-e-N et al., 2021, D-e-N et al., 2023); and a “country meeting”, covering a strengths, weaknesses, opportunities, and threats (SWOT) analysis of HPV prevention and control programs in a particular country or region HPV prevention and control programs (Vorsters et al., 2020, D-e-N et al., 2023).

The 2022 technical meeting of the HPV Prevention and Control Board, held on the 1st and 2nd of June 2022, discussed DNA methylation as a triage tool for cervical cancer screening. Methylation of host gene promotersequences takes place in a wide range of genes, including tumor suppressor genes, histone modification genes, and microRNAs (miRNA) but also in HPV viral genes (Kremer et al., 2021, Albulescu et al., 2021, Dovnik and Poljak, 2023).

Several methods can be used to detect DNA methylation, including quantitative methylation specific polymerase chain reaction (qMSP) (Lo et al., 1999), pyrosequencing (Uhlmann et al., 2002), and whole-genome bisulfite sequencing (Mill et al., 2006). The methods differ in sensitivity, turn-around time, cost, and suitability for screening of large sets of clinical samples.

The objectives for DNA methylation as part of the focused topic meeting were:

• to provide an overview of the potential use of DNA methylation in cervical cancer screening
• to provide an overview of different DNA methylation assays and methods
• to discuss the use of DNA methylation as a novel biomarker for triage of HPV-positive women in cervical cancer screening

## 2 DNA methylation in HPV infection pathophysiology

Most acute HPV infections clear spontaneously or induce low-grade precursor lesions that regress within several months, and less than 10 % eventually progress to high-grade lesions or invasive cancer (Bosch et al., 2008). The development of HPV-related high-grade lesions and cancer is caused by a persistent infection with high-risk HPV genotypes. These lesions are characterised by abnormal expression of the viral oncogenes E6 and E7 in basal and parabasal cells, inducing genomic instability and molecular events that increase the risk of progression to cancer. Novel biomarkers that allow monitoring of the essential molecular events are likely to improve the detection of lesions that have a high risk of progression in both primary screening and triage settings. An accurate prognostic biomarker to distinguish HPV-infected women at risk for progression to cervical intraepithelial neoplasia (CIN) grade 3, and advanced CIN3, i.e., CIN3 with a high cancer risk, or cancer from those who will regress spontaneously without treatment, would change the outline of future cervical cancer screening programmes. Consequently, it would allow more focused interventions on lesions with true potential to progress, reducing repetitive examinations and eliminating treatment for women with regressive CIN.

### 2.1 Overview of different DNA methylation assays used in HPV screening

Several methylation assays were developed targeting host and/or HPV genes, but only a limited number have been extensively tested. Moreover, obtaining consistent performance data across studies has been challenging given the different methodologies used (sample types, target population, thresholds). The QIAsure Methylation Test (Qiagen, Hilden, Germany) which is based on methylation of host genes FAM19A4/miR124-2, showed in four studies, including a large multicentre European trial, a sensitivity for CIN3 + ranging between 69.4 and 77.8 %, and a specificity for < CIN3 ranging between 69.3 and 78.3 % (Vink et al., 2021, Bonde et al., 2021, De Strooper et al., 2016, Leeman et al., 2019). Similarly, the GynTect assay (Oncgnostics, Jena, Germany) based on methylation of six host genes (ASTN1, DLX1, ITGA4, RXFP3, SOX17, and ZNF671) showed in three studies a sensitivity for CIN3 + ranging between 31.6 and 67.7 %, and a specificity for < CIN3 ranging between 82.6 and 95.9 % (Klischke et al., 2021, Schmitz et al., 2018, Schmitz et al., 2017).

In a head-to-head comparison, both GynTect and QIAsure assays detected all CIN3 and cervical carcinoma cases (Dippmann et al., 2020). The differences observed in the detection rates between both assays for the different cervical lesion grades were non-significant.

The S5 classifier targeting the host gene *EPB41L3* and late region L1 genes of four HPV genotypes 16, 18, 31, and 33 (Queen Mary University of London, London, United Kingdom), has also been tested extensively in both high (HIC) and low- and middle-income countries settings (LMICs). Five studies report a sensitivity for CIN3 + ranging between 70.3 and 93.2 % and a specificity for < CIN3 ranging between 35.0 and 76.6 % (Cook et al., 2019, Gu et al., 2020, Hernández-López et al., 2019, van Leeuwen et al., 2019, Ramírez et al., 2021). S5 classifier cut-off for positivity could be adjusted from 0.8 in HIC to 3.7 in LMICs to reflect the local needs and detect only women who need to be referred to colposcopy (Hernández-López et al., 2019, Ramírez et al., 2021, Banila et al., 2022).

## 3 Host and viral DNA methylation markers in HPV-related disease.

### 3.1 Methylation patterns of host cell DNA and the heterogeneous nature of cervical pre-cancer

In a world-wide series of invasive cervical carcinomas from over 25 countries, the QIAsure Methylation Test was used (Vink et al., 2020). In total, 510 of the 519 cervical carcinomas (positivity 98.3 %; 95 % CI: 96.7–99.2 %) tested FAM19A4/miR124-2 methylation positive. Test positivity was consistent across the different subgroups based on cervical cancer histology, ‘Federation Internationale de Gynecologie et d'Obstetrique’ (FIGO) stage, hrHPV (high-risk human papillomavirus genotype) status, sample type (cervical scrapes, fresh frozen, formalin-fixed, paraffin-embedded biopsies) and geographical region (Vink et al., 2020). These results suggest that a negative methylation result could potentially be used to rule out the presence of cervical cancer.

The opportunity of using these biomarkers as a triage tool for cervical pre-cancer (CIN2/3) lesion progression could offer the opportunity to provide more targeted treatment to women with hrHPV infections. An important factor to consider is the heterogeneity in CIN disease, including the variable clinical regression/progression behaviour, the duration of lesion existence, and the diversity of biomarkers present that could signal biological events leading to progression/regression. To test this, 112 cervical scrapes from hrHPV-positive cervical scrapes, covering different underlying histology (no disease, CIN2, CIN3, and cancer) were collected and tested for 12 different host methylation markers. A heterogeneous methylation pattern was observed in CIN2/3 lesions, with 72 % (n = 38/53) of CIN3 and 55 % (n = 11/20) of CIN2, showing a cancer-like methylation-high pattern. This pattern was virtually absent in hrHPV-positive controls with normal cytology (n = 1/26) (Verlaat et al., 2018). To further understand how these biomarkers could predict disease progression, cervical scrapes of hrHPV + women (n = 1,061) < 30 years old were collected and > 30-year-old women were used as reference population (n = 2,264). Methylation analysis included FAM19A4/miR124-2 host genes, and immunohistochemistry stain was carried out using p16ink4a/Ki-67 (transforming HPV infection and cell cycle activity marker, respectively) and HPV E4 stains (productivity marker). FAM19A4/miR124-2 methylation-positive CIN2/3 lesions appeared to be associated with low HPV E4 and high p16ink4a/Ki-67 (non-productive, progression-associated transforming features) expression in young women. This suggests that the subset of women with CIN2 + displaying a cancer-like methylation-high pattern is at higher risk of progression to cancer.

In summary, as methylation-negative CIN2 + cases more frequently show regression (Kremer et al., 2022:Jco2102433., Louvanto et al., 2020), there is a clear clinical value for methylation analysis, as triage in screening or guidance for management.

*Host And Viral DNA Methylation: A Combined Marker Approach.*The S5 classifier is a DNA methylation classifier designed to serve as a triage test for CIN2/3. Using the S5 classifier, 1,493 cervical specimens from a colposcopy cohort were analysed for DNA methylation levels by pyrosequencing (Brentnall et al., 2014). A prediction rule was set to develop a molecular reflex test for triage that maintains the high sensitivity of HPV testing but has better specificity. Diagnostic test results showed an area under the curve (AUC) value of 0.82 (95 % CI: 0.80–0.84), indicating that a methylation assay in hrHPV-positive women might improve the positive predictive value with minimal sensitivity loss.

In the HPV FOCAL (NCT04185389) randomised cervical cancer screening trial, the S5 classifier was tested for its performance to triage hrHPV-positive samples (Cook et al., 2019). Methylation levels increased significantly with disease severity. Relative sensitivity (CIN3) and specificity (<CIN3) of S5 were 93.2 % (95 % CI: 81.4–98.0) and 41.8 % (95 % CI: 35.2–48.8), respectively, compared to 86.4 % (95 % CI: 75.0–95.7) and 49.8 % (95 % CI: 43.1–56.6), for combined abnormal cytology (≥ASCUS) and HPV16/18 positivity (Cook et al., 2019).

## 4 Characterisation of DNA methylation patterns in self-collected samples

### 4.1 The use of DNA methylation in self-collected samples: S5 classifier

In the Predictors 5.1 study which focuses on self-sampling, women referred for colposcopy were invited to provide a first-void urine (FVU) sample collected with the Colli-Pee device (Novosanis, Wijnegem, Belgium) and to take two vaginal self-samples, using either dacron-based digene Female Swab Specimen Collection Kit (Qiagen, Hilden, Germany) and the Copan FLOQswab (Copan Diagnostics, Murrieta, California, US), or a HerSwab (Eve Medical, Toronto, Canada) and Qvintip device (Aprovix AB, Uppsala, Sweden) (Cadman et al., 2021). HPV testing was performed using the BD Onclarity HPV Assay (Becton Dickinson and Company, Franklin Lakes, New Jersey, US). Similar positivity rates and sensitivities for CIN2 + and CIN3 + were seen for the dry flocked swab, the wet Dacron swab, and FVU but lower values were seen for the Qvintip device and the HerSwab. No clear user preferences were seen between devices, but women found urine easiest to collect and were more confident that they had taken the sample correctly (Cadman et al., 2021). This supports the broader use of both urine and vaginal self-sampling for cervical screening. In younger women (<30 years), the clinical sensitivity of methylation testing is much lower than in older women, which may indicate that CIN3 lesions detected in young women are more recent lesions with a high chance of regression.

### 4.2 Self-sampling focused biomarkers: discovery of host markers ASCL1 and LHX8

Self-sampling is a promising strategy to overcome some of the existing barriers to cervical cancer screening, especially in un(der)-screened populations. As of 2020, 17 countries worldwide have implemented self-sampling as part of their national programs (Serrano et al., 2022).

In The Netherlands, the implementation of self-sampling is advocated to target not only non-responders but also be offered to all women within screening age. It has been shown that methylation on vaginal self-samples of hrHPV women (Cervex-Brush, Rovers Medical Devices, Oss, The Netherlands) suspended in 20 ml ThinPrep medium (Hologic, Marlborough, United States) using Roche Cobas 4800 HPV assay (Roche Diagnostics, Indianapolis, United States) has a lower sensitivity (78.4 % versus 88.2 %) for detecting CIN3 + compared to clinician-collected cervical samples (Luttmer et al., 2016). Genome-wide DNA methylation profiling was used to identify a DNA methylation classifier for detection of CIN3+, applicable to lavage and brush-based self-samples, revealing 12 promising DNA methylation markers (Verlaat et al., 2018). Further analysis of these markers in large series of lavage and brush-based self-samples yielded a 3-gene methylation classifier (ASCL1/LHX8/ ST6GALNAC5), with excellent clinical performance for CIN3 detection in both lavage (AUC = 0.88; sensitivity = 74 %; specificity = 79 %) and brush-based self-samples (AUC = 0.90; sensitivity = 88 %; specificity = 81 %) (Verlaat et al., 2018). This classifier for self-samples may be superior to currently available methods.

Data from the Dutch IMPROVE screening trial (NTR5078) showed that individual methylation markers in clinican-collected samples had good performance for CIN3 + detection, with the highest AUC for ASCL1 (0.84) and LHX8 (0.83) (Verhoef et al., 2022). Combined as a bi-marker panel, ASCL1/LHX8 yielded a CIN3 + sensitivity of 76.9 % (70/91; 95 % CI 68.3–85.6 %) at a specificity of 74.5 % (465/624; 95 % CI 71.1–77.9 %). This data indicates that these markers could be optimised for use in self-sampling in a triage setting.

### 4.3 The use of self-collected urine samples for DNA methylation in cervical screening

Why does a methylation test work on urine? During urination, the first part of the urine void, defined as first-void urine (FVU), captures mucus and debris from exfoliated cells from the female genital organs, including the cervix. Alternatively, (pre)cancerous cell DNA circulating in blood might be filtered by the kidney, and fractions may end up in the first fraction of urine (Hentschel et al., 2021). An update of a previously published review of the literature on (bio)markers for cervical cancer in urine was performed on June 1st, 2022 (PubMed, Web of Science) (Van Keer et al., 2017). Twelve papers were withheld after screening 31 individual manuscripts for eligibility. Seven out of twelve studies reported on the use of DNA methylation in urine, including one study focusing on host and viral gene methylation (Guerrero-Preston et al., 2016) and six on host gene methylation only (Feng et al., 2007, Snoek et al., 2019, van den Helder et al., 2022, van den Helder et al., 2020, Van Keer et al., 2021, Hoffstetter et al., 2017). Six studies reported on the clinical performance of DNA methylation using quantitative methylation specific PCR (qMSP) on bisulphite-converted urine DNA (Guerrero-Preston et al., 2016, Snoek et al., 2019, van den Helder et al., 2022, van den Helder et al., 2020, Hoffstetter et al., 2017). Most recent results by van den Helder and colleagues (2022) demonstrate an AUC of 0.84 (86 % sensitivity at 70 % predefined specificity) of the ASCL1/LHX8 bi-marker panel to discern urine samples from women diagnosed with CIN3 + versus healthy controls (van den Helder et al., 2022). The clinical performance of this bi-marker panel will be further evaluated on home-collected first-void urine samples from 458 women aged 24–78 years, with a broad cervical health status spectrum (healthy controls and women diagnosed with CIN1-3 and cancer) (CASUS trial, NCT04530201, METc VUmc: 2020.020) (unpublished data).

However, differences in study design (e.g., study population, urine void, sampling method, DNA preservation, DNA extraction, bisulphite conversion, methylation assay) make comparing clinical accuracy between studies difficult. Therefore, guidelines for validating methylation-based testing strategies in the context of cervical screening are warranted. Notwithstanding that more studies on DNA methylation in urine are needed, we can conclude that this strategy has proven its feasibility for use as a molecular biomarker for cervical screening. Self-collected urine studies showed promising results (Poljak et al., 2023).

## 5 The use of DNA methylation in cervical cancer screening

### 5.1 DNA methylation as a triage test in HPV-positive women

In HPV-based screening programmes, cytology and methylation have similar accuracy for detecting CIN3 + when used as triage. However, the long-term risk of developing cervical cancer is lower in methylation-negative, HPV-positive women than in HPV-positive women with negative cytology (Dick et al., 2019, De Strooper et al., 2018). Another post-hoc study in a Dutch screening trial evaluating 9- and 14-year risk of progressing to CIN3 + for baseline cytology-negative women and baseline FAM19A4/miR124-2 methylation-negative women showed non-significant risk differences of –0.42 % (95 % CI −2.1 to 1.4 %) and −0.07 % (95 % CI −1.9 to 1.9 %), respectively (Vink et al., 2021). In the same study in which women were originally managed based on cytology and HPV, the 14-year cumulative cervical cancer incidence was significantly lower for methylation-negative women compared to cytology-negative women (risk difference 0.98 %, 95 % CI 0.26 to 2.0) between the two triage strategies.

To improve accuracy in identifying HPV-related lesions that may progress to cervical cancer and subsequently lower unnecessary colposcopy referrals, a more effective triage strategy is needed. One study examined the use of FAM19A4/miR124-2 methylation and HPV genotyping in HPV-positive women with borderline or mild dyskaryosis (BMD) to determine the risk of CIN3+ (Dick et al., 2022). The study found that methylation testing had a sensitivity of 70.2 % (95 % CI: 58.3–82.1 %) and specificity 65.8 % (95 % CI: 59.8–71.9 %) with only 3.0 referrals needed to detect one CIN3 + case. Women who tested positive for methylation had a 33.1 % risk of CIN3 + while those who tested negative had a 9.8 % risk. In contrast, women who tested positive for HPV16/18 or HPV16/18/31/33/45 had a 27.6 % or 24.6 % risk of CIN3 + respectively, while those who tested negative had a 13.2 % or 9.1 % risk. Therefore, incorporating methylation testing and/or HPV genotyping into the care of HPV-positive women with BMD can reduce unnecessary colposcopy referrals compared to reflex cytology (Dick et al., 2022). In summary, DNA methylation can be used as a triage test in HPV-positive women with ASCUS/LSIL.

### 5.2 Could DNA methylation be used as a primary cervical screening method?

The evolution of precancerous cervical lesions has been hypothesised to follow the “sequential progression” model in which there is a gradual progression from normal tissue through CIN1 and CIN2 to CIN3. Another hypothesis, the “molecular switch” model, postulates that CIN3 can evolve directly from HPV-infected normal epithelium without progressing through CIN1 and CIN2. Comparing DNA methylation of selected human biomarkers and HPV genotypes in two groups of CIN1, CIN1 that were near or adjacent to CIN3 (adjacent-CIN1) and CIN1 that were the principal lesions with no CIN3 detected (principal-CIN1), showed that adjacent-CIN1 predominantly shared identical HPV genotypes as the CIN3; however, methylation differed substantially between adjacent-CIN1 and CIN3 (Nedjai et al., 2018). In contrast, principal-CIN1 had an indistinguishable methylation distribution compared to adjacent-CIN1 (Nedjai et al., 2018), favouring the “molecular switch” model. A primary screening test strategy using DNA methylation would detect and refer only those high-grade HPV-related lesions with true potential to progress to invasive cervical cancer. Ongoing debates exist on the pragmatism of using methylation assays as a primary cervical screening method. Factors considered include their accuracy in specific populations, affordability, and clinical implementation. The current low specificity of HPV DNA tests results in a high number of unnecessary referrals, which is likely to be further aggravated by the entry of HPV-vaccinated cohorts into cervical screening programs (Lehtinen et al., 2023). A new approach to screening is needed, which involves using molecular triage tests with high sensitivity and specificity and allows for self-sampling. This has been demonstrated by different studies in the previous sections on using self-samples for triage with DNA methylation.

In summary, DNA methylation tests could be used for primary screening, however, optimisation is needed. For this, new international guidelines for using DNA methylation tests as triage and primary screening tests are necessary, in analogy to the Meijer guidelines for HPV DNA testing (Meijer et al., 2009). To be of value in low-resource settings, a point-of-care methylation test must be designed that is reliable, affordable, and mobile.

### 5.3 Methylation for predicting progression of untreated cervical intraepithelial neoplasia

In the Untreated Cervical Intraepithelial Neoplasia, grade 2 (UCIN2) study (Louvanto et al., 2020), young women (18–30 years) with biopsy-confirmed CIN2 were followed up for 24 months with Pap smears, HPV DNA tests, and biopsies if needed. If biopsies reported CIN3, or the patient did not want to continue the study or moved out of the study area, loop electrosurgical excision procedure (LEEP) was performed. Pyrosequencing methylation assays (S5 classifier) were run on exfoliated cervical cells from the Pap smears (Banila et al., 2022, Brentnall et al., 2014). Of 149 women included in the study, 77.8 % were positive for high-risk HPV (hrHPV), and three clinical outcome groups were defined: progression to CIN3 or worse (n = 25); regression to CIN1 or less (n = 88); and persistence in CIN2 (n = 36). Positivity of the S5 classifier was significantly correlated with progression to CIN3. The S5 classifier had a higher sensitivity for CIN3 than cytology (83.6 %; 95 % CI:71.9–91.8 vs 62.3 %; 95 % CI:49.0–74.4) and better positive and negative predictive values than cytology when all measures (cytology and methylation markers) were adjusted for specificity. Using regression of CIN1 or less as the reference, the Odds Ratio (OR) for progression to CIN3 was 3.39 for the S5 classifier and 2.32 for cytology. Incidentally, positivity for HPV genotypes 16, 18, 31 and/or 33 (modified general primer PCR-reverse line blot, Karolinska Institutet, Solna, Sweden) showed a similar OR for progression to CIN3 + and persistence in CIN2. Based on a combination of S5 classifier, HPV16/18 positivity and high-grade squamous intraepithelial lesion or worse (≥HSIL) cytology, more than 50 % of the women who scored positive on all markers progressed, versus less than 10 % of the women who were negative for all markers (p = 0.03) (Louvanto et al., 2020).

A similar observational clinical trial, the CONCERVE study, evaluating the prognostic value of the QIAsure methylation test (Qiagen, Hilden, Germany), targeting the host genes FAM19A4/miR124-2, for clinical regression of CIN2/3 showed that a negative methylation test result on the clinician-collected cervical samples at baseline is associated with clinical regression of CIN2/3 (OR: 2.72; 95 % CI: 1.41–5.26). According to the study, women who had a methylation-negative baseline clinician-collected sample had a significantly higher regression rate of CIN2/3 (74.7 %; 95 % CI: 65.7–81.7) after a 2-year follow-up compared to women with a methylation-positive baseline clinician-collected sample (51.4 %; 95 % CI: 34.6 to 65.9) (P = 0.013) (Kremer et al., 2022). Notably, similar results were obtained on self-collected samples. Regression in women with a negative FAM19A4/miR124-2 methylation test was highest when cytology was atypical squamous cells of undetermined significance (ASCUS)/low-grade squamous intraepithelial lesion (LSIL) (88.4 %) or HPV16 negative (85.1 %).

In summary, methylation assays, alone or in in combination with HPV DNA testing or cytology, have shown to have the potential to be used as triage tools in primary HPV screening and to predict the regression or progression of CIN2/3 lesions.

### 5.4 Evaluation of DNA methylation-based screening strategies in women living with HIV

A prospective observational multicentre cohort study was performed on a South African population of women living with HIV (WLWHIV) to assess the value of hrHPV testing and methylation analysis, alone or in combination, on clinician-collected cervical scrapes to detect CIN3+ (Van Zummeren et al., 2017). Both hrHPV positivity (GP5+/6 + PCR–EIA, DDL, Rijswijk, The Netherlands) and methylation rates of markers CADM1/MAL/ miR124-2 (PreCursor-M, Self-screen B.V., Amsterdam, The Netherlands) increased with severity of cervical disease, reaching 100 % in women with cervical cancer. HrHPV-testing with reflex methylation analysis showed a CIN3 + sensitivity of 73.8 % and a specificity of 81.5 % (Van Zummeren et al., 2017).

In a second South African screening study including 285 WLWHIV, QIAsure Methylation Test was used as a triage strategy (Kremer et al., 2019). The baseline analysis (biopsy confirmed) showed that 68.1 % of the women had no dysplasia or CIN1, 11.3 % had CIN2, 20.0 % had CIN3, and 0.7 % had invasive cervical cancer. Various screening strategies were evaluated, including cytology and HPV-based approaches for detecting CIN3 +. Cytology-based (≥HSIL) strategies had the highest specificities (91.6 % for < CIN3 [95 % CI: 88.0–95.2]) for a single cytology test (no triage). However, it had a low sensitivity, detecting 59.3 % (95 % CI: 46.8–71.9) of CIN3 +. HPV-based (hrHPV) strategies had the highest sensitivities detecting 83.1 % (95 % CI: 73.5–92.6) of CIN3 + for a single hrHPV test. However, it had lower specificities when compared to cytology-based screening (66.4 % [ 95 % CI: 60.2–72.5] vs 91.6 % [95 % CI: 88.0–95.2]).

When combining cytology with FAM19A4/miR124-2 methylation for triage of of women with HSIL-cytology and women with ASCUS/LSIL-cytology, sensitivity was slightly increased (67.8 % [95 % CI: 55.9–79.7) compared to using cytology only (59.3 % [95 % CI: 46.8–71.9]). Cytology and DNA methylation combined strategy had lower specificity (85.0 % [95 % CI:80.3–89.6] in comparison to cytology only (91.6 % [95 % CI: 88.0–95.2]). Combined strategies using HPV testing (triage of 14 high-risk HPV genotypes and individual HPV16/18 genotyping) with FAM19A4/miR124-2 methylation triage provided increased relative sensitivities (cytology ≥ HSIL used as reference) when compared to a single hrHPV test (ratios 1.15 and 1.13, respectively, p-values < 0.001) at the cost of a slight decrease in specificity (ratios 0.88 and 0.96, p-values = 0.03 and 0.5, respectively). Both HPV with FAM19A4/miR124-2 methylation triage of hrHPV positives and HPV16/18 genotyping with FAM19A4/miR124-2 methylation triage of non-HPV16/18 hrHPV positives provided good sensitivities, detecting 72.9 % (95 % CI: 61.5–84.2) and 79.7 % (95 % CI: 69.4–89.9) of CIN3+, respectively, while retaining specificities of 76.1 % (95 % CI: 70.5–81.7) and 74.8 % (95 % CI: 69.1–80.4). In summary, the study demonstrated that HPV-based screening strategies showed higher sensitivity for detecting CIN than cytology-based strategies, albeit with lower specificities. Combining cytology with molecular markers such as FAM19A4/miR124-2 methylation led to modest improvements in sensitivity but at the cost of decreased specificity. A single hrHPV test showed the highest sensitivity for detecting CIN3, but with a higher referral rate and lower specificity. Combined strategies using HPV testing with FAM19A4/miR124-2 methylation triage provided good sensitivities while maintaining acceptable specificities. These findings suggest the potential of methylation markers, particularly in combination with HPV testing, to improve cervical screening outcomes for WLWHIV in LMICs (Kremer et al., 2019).

Finally, in the DIAgnosis in Vaccine And Cervical Cancer Screen (DiaVACCS) trial (Dreyer et al., 2022), a post hoc analysis of ASCL1/LHX8 methylation markers within WLWHIV from the original study again showed a clear increase in methylation with increased disease severity. Methylation in primary screening setting showed a high sensitivity for CIN3 + detection (84.6 % [95 % CI:76.6–92.6]) and high specificity (86.7 % [95 % CI:82.4–91.0) for ≤ CIN1 detection. The bi-marker panel had a higher sensitivity for CIN3 + detection and similar specificity ≤ CIN1 detection as cytology (sensitivity 74 % [95 % CI: 64.2–83.8] and specificity 91 % [95 % CI: 87.3–94.3]), and a slightly lower sensitivity but higher specificity compared to HPV testing (sensitivity 93.6 % [95 % CI: 88.2–99.0] and specificity 78.3 % [95 % CI:73.1–83.5]) (Vink et al., 2022).

In summary, DNA methylation is suitable for primary screening of WLWHIV, offering a full molecular alternative to cytology or HPV-based screening without the need for additional triage testing. See (Table 1).

**Table 1:** DNA Methylation as triage tool for cervical cancer screening: Lessons learned and the way forward.

***Lessons Learned***	***The way forward***
• Methylation level increase with disease progression, making DNA methylation an accurate marker for prediction of disease progression in people infected with high-risk HPV. • Virtually all cervical cancers are methylation positive. • Half of CIN2 and two-thirds of CIN3 cases show a cervical cancer pattern(s). Identification of this pattern can be used as a predictor of progression/regression to invasive cervical cancer. • Multiple studies showed accuracy of methylation assays as a triage tool for HPV-based cervical cancer screening. DNA methylation might be a replacement for cytology as a triage strategy. • After long-term follow-up, methylation-negative women at baseline have lower cumulative cervical cancer incidence than cytology-negative women at baseline. • In women, methylation assays can be performed on both clinician-collected cervical samples and self-collected vaginal and urine samples. • Efficiency of methylation assays varies between sample types. (clinician collected vs. self-collected)	• International guidelines are needed for the validation and use of DNA methylation tests as triage/primary screening test. • There is a need to generate more evidence-based data to support the transition towards triage/primary screening by methylation assays. • Adaptation of methylation assays for use in LMIC is needed (cost, self-sampling device, POC test). • Longitudinal studies using methylation in WLWHIV are needed. So far, only cross-sectional studies have been performed. • Validation studies of different methylation markers are needed in different populations, on different (self-)sample types.

CIN: cervical intraepithelial neoplasia; LMIC: Low-middle income country; POC: Point-of-care; WLWHIV: Women living with HIV.

## 6 Novel DNA methylation biomarkers

### 6.1 HPV DNA methylation as biomarker for cervical pre-cancer: consistency across 12 genotypes and potential impact on the management of HPV-positive women

In HPV-based screening, extended HPV genotyping gives information about the individual risk of the patient to develop neoplasia and insights into how common each genotype is. HPV16 is both high-risk and common, whereas other HPV genotypes have a lower risk and are less common. In any case, HPV genotyping alone cannot distinguish between HPV transient infections and precancerous lesions. Increased HPV L1 methylation is observed in pre-cancer in the four most common carcinogenic genotypes (Clarke et al., 2012, Mirabello et al., 2012, Wentzensen et al., 2012). Moreover, methylation shows which genotype is the causal genotype in women with multiple infections.

Next-generation bisulphite sequencing for 12 carcinogenic HPV genotypes (HPV 16, 18, 31, 33, 35, 39, 45, 51, 52,56, 58, and 59) was performed on CpG sites (10–15 sites per genotype) within the late regions of hrHPV genes (L1 and L2), showing that HPV DNA methylation is a general phenomenon marking the transition from HPV infection to pre-cancer (Clarke et al., 2018). Methylation was positively associated with CIN3/Adenocarcinoma *in situ* (AIS) across all 12 genotypes. AUCs for the top sites ranged from 0.71 (HPV51 and HPV56) to 0.86 (HPV18). The 12-genotype methylation assay (sensitivity fixed at 80 %) had higher specificity (65.5 % vs. 54.1 %) and lower test positivity (38.5 % vs. 48.7 %) compared with cytology detecting ASCUS +. Comparing triage algorithms, the risk of CIN3/AIS was highest for methylation positives and lowest for cytology or HPV16/18 positives (Clarke et al., 2018). Using 12-genotype methylation assay, it is possible to detect HPV status, HPV genotype, and methylation status, all from the same sample. The test is also suitable for self-collected samples, is amenable to automation and provides a quantitative result, which allows tailoring thresholds to different settings. Further sample sites (anal, cervical), sample strategy (clinician collected, self-collected) and population (African American woman, cisgender, transgender, people living with HIV) validation are under study.

In summary, the value of DNA methylation for triage is not dependent on the HPV genotype involved but in the prognosis of these molecular events leading to pre-cancer.

## 7 Discussion

Studies that yielded similar accuracy results in the performance of DNA methylation versus cytology for CIN3 + detection in HPV-positive women mostly occurred in settings that have cytology of optimal quality. However, if the performance of DNA methylation versus cytology had been evaluated in settings with cytology of suboptimal quality, the results would have been substantially different e.g., showing inferiority of cytology. Namely, while cytology accuracy varies in different settings, DNA methylation is an objective and reproducible molecular triage test providing accurate results across settings. Furthermore, in settings without comprehensive cervical cancer screening programs, methylation alone could be used for primary screening, referring HPV-positive/methylation positive women to immediate treatment and negative women to follow-up after an appropriate number of years using a single sample. Validation of methylation tests needs to include sampling tools that can be used locally. The question on how these tests can be implemented in local laboratories focused on procurement price of HPV/methylation tests. Thus, affordable alternatives are needed. Since the price of tests and devices is mainly driven by volume, there is hope that the cost per test could decrease if the volume of testing significantly increases. Another concern is that of DNA degradation during transport when using dry samples. Finally, there is a need for high-throughput methylation tests on the market. While acknowledging the limitations of the studies summarized in this manuscript, it's important to note that multiple large post-hoc studies in prospective screening trials have been performed QIAsure and S5. Additionally, prospective validation studies have been conducted for the management of CIN2/3 patients, yet there remains a need for more evidence-based data. Nonetheless, it isimportant to recognise that the existing data is invaluable in continuing the research on DNA methylation in cervical cancer screening. This research is crucial to further extend the evaluation of methylation as a triage strategy effectively but also to aid in its transition towards triage/primary screening, and to inform the development of international validation guidelines that address technical aspects such as test specifications (including predefined cut-offs), test reproducibility (e.g. as shown for QIAsure – (Floore et al., 2019) and handling of invalid samples. As a short-term strategy, self-sampling implementation should be prioritised in LMICs.

In HICs, the transition to methylation is expected to go more smoothly and timely. In The Netherlands, where women are offered primary screening via clinician-collected or self-collected samples, using methylation as triage could reduce the need to visit the general practitioner for a Pap smear to perform reflex triage cytology.

In all settings, follow-up after a positive screen test remains essential. A risk-based, context-adjusted system is needed to decide what to do with methylation-positive/HPV-positive, methylation-positive/HPV-negative, methylation-negative/HPV-positive and methylation-negative/HPV-negative results.

For WLWHIV, current cervical cancer screening strategies in primary and triage settings are suboptimal with variable sensitivities across studies. DNA methylation has shown to be a promising strategy.

## 8 Consent for publication

HPV Prevention and Control Board meetings are invitation-only meetings. All participants accepted the invitation and attended the meeting out of their free will. The HPV Prevention and Control Board asked the participants to fill out a ‘consent form’, agreeing that the videos and photos of the meetings can be published online. The speakers are also asked to fill out a consent form to agree/disagree that their presentation can be published on the website, included in the meeting report or used for publication.

## 9 Ethical compliance

The corresponding author warrants that if the manuscript describes research on human subjects the necessary ethical approval (or exemption) has been obtained and is on file with the authors’ institutions. For empirical research papers, add a statement of ethical compliance or exemption to the Methods section.

## 10 Authorship and originality

The corresponding author warrants that all aforementioned authors fulfill the criteria of authorship as defined by the International Committee of Medical Journal Editors (ICMJE). The corresponding author further warrants that the work described in this manuscript has not been published before and is not (nor will be) under consideration elsewhere while under review in *Preventive Medicine Reports*; that all authors approved the present submitted version and their institutions have no objections to the manuscript’s contents.

## Funding

The HPV Prevention and Control Board is supported by in kind contributions and support from the international experts involved and their institutions. To set up the activities and support publication costs, the secretariat obtained unrestricted grants from industry (GlaxoSmithKline Biologicals, Merck). All funds were handled according to the rules of the University of Antwerp. No remuneration for experts or speakers was provided.

## CRediT authorship contribution statement

**F. Ricardo Burdier:** Writing – review & editing, Project administration, Formal analysis, Data curation, Conceptualization. **Dur-e-Nayab Waheed:** Writing – review & editing, Project administration, Formal analysis, Data curation, Conceptualization. **Belinda Nedjai:** Writing – review & editing. **Renske D.M. Steenbergen:** Writing – review & editing. **Mario Poljak:** Writing – review & editing. **Marc Baay:** Writing – review & editing, Writing – original draft. **Alex Vorsters:** Writing – review & editing, Project administration, Investigation, Formal analysis, Data curation, Conceptualization. **Severien Van Keer:** Writing – review & editing, Writing – original draft, Methodology, Investigation, Data curation, Conceptualization.

## Declaration of competing interest

The authors declare the following financial interests/personal relationships which may be considered as potential competing interests: AV University of Antwerp obtained unrestricted educational grants from GSK, Merck, Roche and Hologic; an investigator-initiated grant from Merck and speaker fees from Merck. RDMS is a minority shareholder of Self-screen BV. Self-screen holds patents and products related to the work described. MP’s institution received research funding, free-of-charge reagents, and consumables to support HPV methylation research in the last 3 years from Qiagen, all paid to his employer. MB received medical writing fees from Merck, SPMSD and GSK. SVK is a member of the Scientific Advisory Board (SAB) on Methylation of Novosanis since 2022. Alex Vorsters reports his institution has received financial support from Bill and Melinda Gates Foundation. Alex Vorsters reports a relationship between his institute Center for the Evaluation of Vaccination, Faculty of Medicine and Health Sciences, University of Antwerp and GlaxoSmithKline Biologicals, Merck, Roche Diagnostics, Becton Dickinson, and Hologic that includes: funding grants. DNW and FRB have no known competing financial interests or personal relationships that could have appeared to influence the work reported in this paper.

## Data Availability

All the presentations of the meeting report are published on the website (https://www.hpvboard.org) after speakers’ approval.
